# Prediction of Protein–Protein Interaction with Pairwise Kernel Support Vector Machine

**DOI:** 10.3390/ijms15023220

**Published:** 2014-02-21

**Authors:** Shao-Wu Zhang, Li-Yang Hao, Ting-He Zhang

**Affiliations:** 1 College of Automation, Northwestern Polytechnical University, Xi’an 710072, China; E-Mails: haoliyang0306@sina.com (L.-Y.H.); zth86z68@163.com (T.-H.Z.); 2 Key Laboratory of Information Fusion Technology, Ministry of Education, Xi’an 710072, China

**Keywords:** amino acid distance frequency, amino acid index distribution, protein-protein interaction, pairwise kernel function, support vector machine

## Abstract

Protein–protein interactions (PPIs) play a key role in many cellular processes. Unfortunately, the experimental methods currently used to identify PPIs are both time-consuming and expensive. These obstacles could be overcome by developing computational approaches to predict PPIs. Here, we report two methods of amino acids feature extraction: (i) distance frequency with PCA reducing the dimension (DFPCA) and (ii) amino acid index distribution (AAID) representing the protein sequences. In order to obtain the most robust and reliable results for PPI prediction, pairwise kernel function and support vector machines (SVM) were employed to avoid the concatenation order of two feature vectors generated with two proteins. The highest prediction accuracies of AAID and DFPCA were 94% and 93.96%, respectively, using the 10 CV test, and the results of pairwise radial basis kernel function are considerably improved over those based on radial basis kernel function. Overall, the PPI prediction tool, termed PPI-PKSVM, which is freely available at http://159.226.118.31/PPI/index.html, promises to become useful in such areas as bio-analysis and drug development.

## 1. Introduction

Protein–protein interactions (PPIs) play an important role in such biological processes as host immune response, the regulation of enzymes, signal transduction and mediating cell adhesion. Understanding PPIs will bring more insight to disease etiology at the molecular level and potentially simplify the discovery of novel drug targets [1]. Information about protein–protein interactions have also been used to address many biological important problems [2–5], such as prediction of protein function [2], regulatory pathways [3], signal propagation during colorectal cancer progression [4], and identification of colorectal cancer related genes [5]. Experimental methods of identifying PPIs can be roughly categorized into low- and high-throughput methods [6]. However, PPI data obtained from low-throughput methods only cover a small fraction of the complete PPI network, and high-throughput methods often produce a high frequency of false PPI information [7]. Moreover, experimental methods are expensive, time-consuming and labor-intensive. The development of reliable computational methods to facilitate the identification of PPIs could overcome these obstacles.

Thus far, a number of computational approaches have been developed for the large-scale prediction of PPIs based on protein sequence, structure and evolutionary relationship in complete genomes. These methods can be roughly categorized into those that are genomic-based [8,9], structure-based [10], and sequence-based [11–26]. Genomic- and structure-based methods cannot be implemented if prior information about the proteins is not available. Sequence-based methods are more universal, but they concatenate the two feature vectors of protein *P_a_* and *P_b_* to represent the protein pair *P_a_*–*P_b_*, and the concatenation order of two feature vectors will affect the prediction results. For example, if we use feature vectors *x_a_*, *x* *_b_* to represent protein *P_a_* and *P_b_*, respectively, then the *P_a_*–*P_b_* protein pair can be expressed as *x_ab_* = *x_a_* ⊕ *x_b_*, or *x_ba_* = *x_b_* ⊕ *x_a_*. In general, however, *x_a_* ⊕ *x_b_* is not equal to *x_b_* ⊕ *x_a_*. Furthermore, PPIs have a symmetrical character; that is, the interaction of protein *P_a_* with protein *P_b_* equals the interaction of protein *P_b_* with protein *P_a_*. Under these circumstances, concatenating two feature vectors of protein *P_a_* and *P_b_* to represent the protein pair *P_a_*–*P_b_* and then using the traditional kernel *k*(*x*_1_, *x*_2_) to predict PPIs would not be workable.

Therefore, in this paper, we introduced two kinds of feature extraction approaches, amino acid distance frequency with PCA reducing the dimension (DFPCA) and amino acid index distribution (AAID) to represent the protein sequences, followed by the use of pairwise kernel function and SVM to predict PPI.

## 2. Results and Discussion

LIBSVM [27], loaded from http://www.csie.ntu.edu.tw/~cjlin, is a library for Support Vector Machines (SVMs), and it was used to design the classifier in this paper. The kernel program of the software was modified to the pairwise kernel functions, which were formed by the RBF genomic kernel function *K* (*x*_1_, *x*_2_) in all experiments.

### 2.1. The Results of DFPCA and AADI with K_II_ Pairwise Kernel Function SVM

In statistical prediction, the following three cross-validation methods are often used to examine a predictor for its effectiveness in practical application: independent dataset test, *K*-fold crossover or subsampling test, and jackknife test [28]. However, of the three test methods, the jackknife test is deemed the least arbitrary that can always yield a unique result for a given benchmark dataset as demonstrated by Equations (28)–(30) in [29]. Accordingly, the jackknife test has been increasingly and widely used by investigators to examine the quality of various predictors (see, e.g., [30–41]). However, to reduce the computational time, we adopted the 10-fold cross-validation (10 CV) test in this study as done by many investigators with SVM as the prediction engine.

The four feature vector sets, Hf, Vf, Pf, and Zf, extracted with DFPCA and the five feature vector sets, LEWP710101, QIAN880138, NADH010104, NAGK730103 and AURR980116, extracted with AAID were employed as the input feature vectors for *K*_II_ pairwise radial basis kernel function (PRBF) SVM. The results of DFPCA and AAID are summarized in Table 1.

From Table 1, we can see that the performances of the two feature extraction approaches, *i.e*., amino acid distance frequency with PCA (DFPCA) and amino acid index distribution (AAID), are nearly equal when using the K_II_ pairwise kernel SVM. The total prediction accuracies are 93.69%~94%. As previously noted, we used just five amino acid indices, including LEWP710101, QIAN880138, NADH010104, NAGK730103 and AURR980116, to produce the feature vector sets. When we tested the performance of AAID against the remaining 480 amino acid indices from AAindex, we found that the amino acid index does affect predictive results and that the total prediction accuracies of those amino acid indices were 79.4%~94%. Among our original five indices, as noted above, the performance of AAID was superior in comparison to the results from AAindex. To account for the better performance of our five indices, we point to the physicochemical and biochemical properties of amino acids. By single-linkage clustering, one of agglomerative hierarchical clustering methods, Tomii and Kanehisa [42] divided the minimum spanning of these amino acid indices into six regions: α and turn propensities, β propensity, amino acid composition, hydrophobicity, physicochemical properties, and other properties. The indices of LEWP710101, QIAN880138, NAGK730103 and AURR980116 are arranged into the region of α and turn propensities, while NADH010104 is arranged into the hydrophobicity region, indicating that the properties of α and turn propensities, and hydrophobicity contain more distinguishable information for predicting PPIs.

### 2.2. The Comparison of Pairwise Kernel Function with Traditional Kernel Function

In order to evaluate the performance of pairwise kernel function, we compared the results of pairwise radial basis kernel function (PRBF) and radial basis function kernel (RBF) with the same feature vector sets. For RBF, we concatenate the two feature vectors of protein *P_a_* and protein *P_b_* to represent the protein pair *P_a_* *– P_b_*; that is, feature vector *x_ab_* = *x_a_* ⊕ *x_b_* was used as the input feature vector of RBF. The results of RBF and PRBF with DFPCA in the 10CV test are listed in Table 2.

Table 2 shows that the performance of PRBF is superior to that of RBF for predicting PPI. The total prediction accuracies of PRBF are higher at 3.9%~4.48% than those of RBF.

### 2.3. The Comparison of DF and DFPCA Feature Extraction Approaches

For the feature extraction approach of distance frequency of amino acids grouped with their physicochemical properties, we compared the results of DF and DFPCA with PRBF SVM to test the validity of adopting PCA. The reduced feature matrix is set to retain 99.9% information of the original feature matrix by PCA. The results of DF and DFPCA with PRBF SVM in the 10CV test are listed in Table 3.

From Table 3, we can see that the performance of DFPCA is superior to that of DF. The total prediction accuracies and *MCC* (see Equation (16) below) of DFPCA are 15.79%~24.43% and 0.2705~0.4067 higher than those of DF, respectively. Although the sensitivities of DF are a little higher (1.43%~1.59%) than those of DFPCA for the Hf, Vf, Pf and Zf feature sets, the positive predictive values are much less than that of DFPCA (21%~29%), which means that the DFPCA approach can largely reduce the false positives. These results show that the performance of DFPCA is superior to that of DF for predicting PPI. It should be noted that feature vectors generated with either DF or DFPCA contain statistical information of amino acids in protein sequences, as well as information about amino acid position and physicochemical properties.

### 2.4. The Performance of the Predictive System Influenced by Randomly Sampling the Noninteracting Protein Subchain Pairs

To investigate the influence of randomly sampling the noninteracting protein subchain pairs, we randomly sampled 2510 noninteracting protein subchain pairs five times to construct five negative sets, and we used the DFPCA approach with hydrophobicity property to predict PPI in the 10CV test. The results, as shown in Table 4, indicate that random sampling of the noninteracting protein subchain pairs in order to construct negative sets has little influence on the performance of the PPI-PKSVM.

### 2.5. Comparison of Different Prediction Methods

To demonstrate the prediction performance of our method, we compared it with other methods [25] on a nonredundant dataset constructed by Pan and Shen [25], in which no protein pair has sequence identity higher than 25%. The number of positive links, *i.e*., interacting protein pairs, is 3899, which is composed of 2502 proteins, and the number of negative links, *i.e*., noninteracting protein pairs, is 4262, which is composed of 661 proteins. Among the prediction results of different methods shown in Table 5, the performance of PPI-PKSVM stands out as the best. When compared to Shen’s LDA-RF, the accuracy (see Equation (15) below) and *MCC* of LEWP710101/QIAN880138 and Hf-DFPCA are respectively 1.9%, 2%, 0.038 and 0.039 higher. These results indicate that our method is a very promising computational strategy for predicting protein–protein interaction based on the protein sequences.

## 3. Experimental Section

### 3.1. Dataset

To construct the PPI dataset, we first obtained the subchain pair name of PPIs from the PRISM (Protein Interactions by Structural Matching) server (http://prism.ccbb.ku.edu.tr/prism/), which was used to explore protein interfaces, and we downloaded the corresponding sequences of these protein subchain pairs from the Protein Data Bank (PDB) database (http://www.rcsb.org/pdb/). According to PRISM [43], a subchain pair is defined as an interacting subchain pair if the interface residues of two protein subchains exceed 10; otherwise, the subchain pair is defined as a noninteracting subchain pair. For example, suppose a protein complex has A, B, C and D subchains. If the interface residues of AB, AC, and BD subchain pairs total more than 10, while the interface residues of AD, BC and CD subchain pairs total less than 10, then the AB, AC, and BD subchain pairs are treated as interacting subchain pairs, while the AD, BC and CD subchain pairs are treated as noninteracting subchain pairs. All interacting protein subchain pairs were used in preparing the positive dataset, and all noninteracting subchain pairs were used in preparing the negative dataset. To reduce the redundancy and homology bias for methodology development, all protein subchain pairs were screened according to the following procedures [15]. (i) Protein subchain pairs containing a protein subchain with fewer than 50 amino acids were removed; (ii) For subchain pairs having ≥40% sequence identity, only one subchain pair was kept. The ≥40% determinant may be understood as follows. Suppose protein subchain pair A is formed with protein subchains A1 and A2 and protein subchain pair B is formed with protein subchains B1 and B2. If sequence identity between protein subchains A1 and B1 and A2 and B2 is ≥40%, or sequence identity between protein subchains A1 and B2 and between A2 and B1 is ≥40%, then the two protein subchain pairs are defined as having ≥40% sequence identity. In our method, we would only retain those subchain pairs having <40% sequence identity. After these screening procedures, the resultant positive set was comprised of 2510 interacting protein subchain pairs, while the resultant negative set contained many noninteracting protein subchain pairs. To avoid unbalanced data between the positive and negative sets, we randomly sampled the 2510 noninteracting protein subchain pairs to construct the negative set. Finally, a PPI dataset consisting of 2510 PPI subchain pairs and 2510 noninteracting protein subchain pairs was constructed.

### 3.2. Distance Frequency of Amino Acids Grouped with Their Physicochemical Properties

The frequency of the distance between two successive amino acids, or distance frequency, was used to predict subcellular location by Matsuda *et al*., [44] and can be described as follows: For a protein sequence *P*, the distance set *d_A_* between two successive letters (e.g., *A*) appearing in protein sequence *P* can be represented as:

(1) $$d_{A}=\{d_{1},d_{2},\ldots ,d_{i},\ldots d_{n_{A}-1}\}\;\;\;i=1,\ldots n_{A}-1$$

where *n_A_* is number of letter *As* appearing in protein sequence *P*, *d_i_* is the distance from the *i*th letter *A* to the (*i* + 1)th letter *A*, and *d_i_* is calculated in a left-to-right fashion. The distance frequency vector for letter *A* can be defined by the following equation:

(2) $$f_{A}=[N_{1},N_{2},\cdots ,N_{j},\cdots N_{m}]$$

where *N_j_* represents the number of times that the *j*th distance unit appears in the *d_A_* set. For example, considering the protein sequence *AACDAMMADA*, the distance sets of letters *A*, *C*, *D* and *M* are shown respectively as

$$d_{A}=\{1,3,3,2\},d_{C}=\{0\},d_{D}=\{5\},d_{M}=\{1\}$$

As a result, the corresponding distance frequency vectors are shown respectively as *Df_A_* = [1,1,2,0,0], *Df_C_* = [0,0,0,0,0], *Df_D_* = [0,0,0,0,1], *Df_M_* = [1,0,0,0,0]. The other 16 basic amino acid distance frequency vectors are zero vector, or *V* = [0,0,0,0,0]. Thus, we can use the feature vector *x* to encode the protein sequence *P*:

$$x=[{Df}_{A},{Df}_{C},{Df}_{D},\cdots ,{Df}_{Y}]$$

In this work, we used the concept of distance frequency [44] and borrowed Dubchak’s idea of representing the amino acid sequence with four physicochemical properties [45] to encode the protein subchain sequence. First, according to the amino acid value given by such physicochemical properties as hydrophobicity [46], normalized van der Waals volume [47], polarity [48] and polarizability [49], the 20 natural amino acids can be divided into three groups [45], as listed in the Table 6. For Hydrophobicity, Normalized van der Waals Volume, Polarity and Polarizability, the amino acids in Group 1, Group 2 and Group 3 were expressed as *H*_1_, *H*_2_, *H*_3_; *V*_1_, *V*_2_, *V*_3_; *P*_1_, *P*_2_, *P*_3_; and *Z*_1_, *Z*_2_ and *Z*_3_, respectively. Second, each protein subchain sequence was then translated into the appropriate three-symbol sequence, depending on the particular physicochemical property, be it *H*_1−3_, *V*_1−3_, *P*_1−3_, or *Z*_1−3_. For example, suppose that the original protein sequence is MKEKEFQSKP. Then, by the set of symbols denoted above, in this case, hydrophobicity, this sequence can be translated into *H*_3_*H*_1_*H*_1_*H*_1_*H*_1_*H*_3_*H*_1_*H*_2_*H*_1_*H*_2_, and the same would be true for *V*_1–3_, *P*_1–3_, or *Z*_1–3_. Third, the distance frequency of every symbol in the translated sequence was computed. In the above example, the *H*_1_, *H*_2_, *H*_3_ distance frequency would be respectively computed for the sequence *H*_3_*H*_1_*H*_1_*H*_1_*H*_1_*H*_3_*H*_1_*H*_2_*H*_1_*H*_2_. Finally, every protein subchain sequence can be encoded by the following feature vector:

(3) $$x_{H}={\left[x_{H_{1}},x_{H_{2}},x_{H_{3}}\right]}^{T},x_{V}={\left[x_{V_{1}},x_{V_{2}},x_{V_{3}}\right]}^{T},x_{P}={\left[x_{P_{1}},x_{P_{2}},x_{P_{3}}\right]}^{T},x_{Z}={\left[x_{Z_{1}},x_{Z_{2}},x_{Z_{3}}\right]}^{T}$$

Conveniently, the feature set based on hydrophobicity, normalized van der Waals volume, polarity, and polarizability can be written as Hf, Vf, Pf and Zf, respectively. In general, the dimensions of two feature vectors generated separately by two protein subchains are unequal. To solve this issue, we enlarge the feature vector dimension of one protein subchain such that it has a feature vector dimension equal to that of another subchain. For example, given the following protein subchain pair *P_a_* − *P_b_*:

Subchain *P_a_* amino acid sequence: MKEKEFQSKP
Subchain *P_b_* amino acid sequence: QNSLALHKVIMVGSG

If we adopt the property of hydrophobicity, then *P_a_* and *P_b_* amino acid sequences can be translated into the following symbol sequence, respectively.

Subchain *P_a_*: *H*_3_*H*_1_*H*_1_*H*_1_*H*_1_*H*_3_*H*_1_*H*_2_*H*_1_*H*_2_
Subchain *P_b_*: *H*_1_*H*_1_*H*_2_*H*_3_*H*_2_*H*_3_*H*_2_*H*_1_*H*_3_*H*_3_*H*_3_*H*_3_*H*_2_*H*_2_*H*_2_

Then, the distance sets of subchains *P_a_* and *P_b_* are shown as: $d_{H_{1}}^{a}=\{1,1,1,2,2\},d_{H_{2}}^{a}=\{2\},d_{H_{3}}^{a}=\{5\},d_{H_{1}}^{b}=\{1,6\},d_{H_{2}}^{b}=\{2,2,6,1,1\},d_{H_{3}}^{b}=\{2,3,1,1,1,\}$, and the distance frequency vectors of subchains *P_a_* and *P_b_* are as follows:

$$x_{a}=[x_{H_{1}}^{a},x_{H_{2}}^{a},x_{H_{3}}^{a}],x_{b}=[x_{H_{1}}^{b},x_{H_{2}}^{b},x_{H_{3}}^{b}]$$

where

$$\begin{array}{l} x_{H_{1}}^{a}=[3,2,0,0,0,0],x_{H_{2}}^{a}=[0,1,0,0,0,0],x_{H_{3}}^{a}=[0,0,0,0,1,0],\\ x_{H_{1}}^{b}=[1,0,0,0,0,1],x_{H_{2}}^{b}=[2,2,0,0,0,1],x_{H_{3}}^{b}=[3,1,1,0,0,0] \end{array}$$

Hereinafter we will use “DF” to represent the distance frequency method by grouping amino acids with their physicochemical properties.

By our use of DF to represent the protein subchain pair, we can see that the feature vector is sparse, while the vector dimension is large, when the subchain sequence is longer. To further extract the features, Principal Component Analysis (PCA) was then used to reduce the dimension, and amino acid distance frequency combined with PCA reducing the dimension is now termed DFPCA.

### 3.3. Amino Acid Index Distribution (AAID)

Let *I*_1_, *I*_2_, …, *I_i_*, ···, *I*_20_ be the amino acid physicochemical value of the 20 natural amino acids *α_i_* (A, C, D, E, F, G, H, I, K, L, M, N, P, Q, R, S, T, V, W, and Y), respectively, which can be accessed through the DBGET/LinkDB system by inputting an amino acid index (e.g., LEWP710101). An amino acid index is a set of 20 numerical values representing any of the different physicochemical and biochemical properties of amino acids. We can download these indices from the AAindex database (http://www.genome.jp/aaindex/).

For a given protein sequence *P* whose length is *L*, we replace each residue in the primary sequence by its amino acid physicochemical value, which results in a numerical sequence *h*_1_, *h*_2_, …, *h_l_*, …, *h_L_*, (*h_l_* ∈ *I*_1_, *I*_2_,…, *I*_20_).

Then, we can define the following feature *w_i_* of amino acid *α_i_* to represent the protein sequences:

(4) $$w_{i}=I_{i}•f_{i}$$

Where *f_i_* is the frequency of amino acid *α_i_* that occurs in protein sequecne *P*, *I_i_* is the physicochemical value of amino acid *α_i_*, and the symbol • indicates the simple product. *f_i_* and *I_i_* are mutually independent. Obviously, *w_i_* includes the physicochemical information and statistical information of amino acid *α_i_*, but it loses the sequence-order information. Therefore, to let feature vectors contain more sequence-order information, we introduced the 2-order center distance *d_i_* by considering the position of amino acid *α_i_*, which is defined as

(5) $$d_{i}=\sum_{j=1}^{N_{\alpha_{i}}}{\left(\frac{k_{i,j}-{\bar{k}}_{i}}{L}•I_{i}\right)}^{2}$$

where *N_α_*_*_i_*_ is the total number of amino acid *α_i_* appearing in the protein sequence *P*, *k_i_*_,_ *_j_* (*j* = 1,2, ···, *N_α_*_*_i_*_) is the *j*th position of the amino acid *α_i_* in the sequence, and *k̄_i_* is the mean of the position of amino acid *α_i_*.

Now feature *d_i_* contains the physicochemical information, statistical information and the sequence-order information of amino acid *α_i_*, but it still does not distinguish the protein pairs in some cases. For example, assume two protein pairs *P_a_* – *P_b_* and *P_c_* – *P_d_*. The sequences of protein *P_a_*, *P_b_*, *P_c_* and *P_d_* are respectively shown as:

*P_a_*: MPPRNKPNRR; *P_b_*: MPNPRNNKPPGRKTR
*P_c_*: MPRRNPPNRK; *P_d_*: MGTRPPRNNKPNPRK

Obviously, *P_a_* and *P_c_*, as well as *P_b_* and *P_d_*, have the same *w_i_* and *d_i_*. If we use the orthogonal sum vector, we cannot distinguish between the *P_a_* − *P_b_* and *P_c_* − *P_d_* protein pairs. To solve this problem, the 3-order center distance *t_i_* of amino acid *α_i_* was introduced, which is defined as

(6) $$t_{i}=\sum_{j=1}^{N_{\alpha_{i}}}{\left(\frac{k_{i,j}-{\bar{k}}_{i}}{L}•I_{i}\right)}^{3}$$

Finally, we can use a combined feature vector to represent protein sequence *P* by serializing above three features as

(7) $$x={\left[w_{1},\cdots ,w_{i},\cdots ,w_{20},d_{1},\cdots ,d_{i},\ldots ,d_{20},t_{1},\cdots ,t_{i},\cdots ,t_{20}\right]}^{T}$$

The protein pair *P_a_* – *P_b_* can now be represented by the following feature vectors:

(8) $$x_{ab}={\left[w_{1}^{a},\cdots ,w_{20}^{a},d_{1}^{a},\cdots d_{20}^{a},t_{1}^{a},\cdots ,t_{20}^{a},w_{1}^{b},\cdots ,w_{20}^{b},d_{1}^{b},\cdots ,d_{20}^{b},t_{1}^{b},\cdots ,t_{20}^{b}\right]}^{T}$$

or

(9) $$x_{ba}={\left[w_{1}^{b},\cdots ,w_{20}^{b},d_{1}^{b},\cdots d_{20}^{b},t_{1}^{b},\cdots ,t_{20}^{b},w_{1}^{a},\cdots ,w_{20}^{a},d_{1}^{a},\cdots ,d_{20}^{a},t_{1}^{a},\cdots ,t_{20}^{a}\right]}^{T}$$

Generally, vector *x_ab_* is not equal to vector *x_ba_*. As such, if a query protein pair *P_a_* *– P_b_* is represented by *x_ab_* and *x_ba_* respectively, the prediction results may be different. In this paper, we will choose the pairwise kernel function to solve this dilemma.

### 3.4. Pairwise Kernel Function

Ben-Hur and Noble [13] first introduced a tensor product pairwise kernel function *K*_I_ to measure the similarity between two protein pairs. The comparison between a pair (*x*_1_, *x*_2_) and another pair (*x*_3_, *x*_4_) for *K*_I_ is done through the comparison of *x*_1_ with *x*_3_ and *x*_2_ with *x*_4_, on the one hand, and the comparison of *x*_1_ with *x*_4_ and *x*_2_ with *x*_3_, on the other hand, as

(10) $$K_{\text{I}}((x_{1},x_{2}),(x_{3},x_{4}))=K(x_{1},x_{3})\cdot K(x_{2},x_{4})+K(x_{1},x_{4})\cdot K(x_{2},x_{3})$$

However, the *K*_I_ kernel does not consider differences between the elements of comparison pairs in the feature space; therefore, Vert [50] proposed the following metric learning pairwise kernel *K*_II_:

(11) $$K_{\text{II}}((x_{1},x_{2}),(x_{3},x_{4}))={\left(K(x_{1},x_{3})+K(x_{2},x_{4})-K(x_{1},x_{4})-K(x_{2},x_{3})\right)}^{2}$$

In particular, two protein pairs might be very similar for the *K*_II_ kernel, even if the patterns of the first protein pair are very different from those of the second protein pair, whereas the *K*_I_ kernel could result in a large dissimilarity between the two protein pairs. It is easy to prove that the *K*_II_ kernel satisfies both Mercer’s condition and the pairwise kernel function condition. In this paper, we use the *K*_II_ kernel function to predict PPI.

### 3.5. Assessment of Prediction System

Sensitivity (*S_n_*), specificity (*S_p_*), positive predictive value (*PPV*) and total prediction accuracy (*ACC*) [39–41] were employed to measure the performance of PPI-PKSVM.

(12) $$S_{n}=\frac{TP}{TP+FN}$$

(13) $$S_{p}=\frac{TN}{TN+FP}$$

(14) $$PPV=\frac{TP}{TP+FP}$$

(15) $$ACC=\frac{TP+TN}{TP+TN+FP+FN}$$

(16) $$MCC=\frac{TP\times TN-FP\times FN}{\sqrt{\left(TP+FN)(TP+FP)(TN+FN)(TN+FP\right)}}$$

where *TP* and *TN* are the number of correctly predicted subchain pairs of interacting proteins and noninteracting proteins, respectively, and *FP* and *FN* are the number of incorrectly predicted subchain pairs of noninteracting proteins and interacting proteins, respectively.

## 4. Conclusions

In this work, we introduced two feature extraction approaches to represent the protein sequence. One is amino acid distance frequency with PCA reducing the dimension, termed DFPCA. Another is amino acid index distribution based on the physicochemical values of amino acids, termed AAID. The pairwise kernel function SVM was employed as the classifier to predict the PPIs. From the results, we can conclude that (i) the performance of DFPCA is better than that of DF; (ii) the prediction power of PRBF is superior to RBF, suggesting that designing a rational pairwise kernel function is important for predicting PPIs; (iii) DFPCA and AAID with pairwise kernel function SVM are effective and promising approaches for predicting PPIs and may complement existing methods. Since user-friendly and publicly accessible web servers represent the future direction in the development of predictors, we have provided a web server for PPI-PKSVM, and it can be found at (http://159.226.118.31/PPI/index.html). PPI-PKSVM in its present version can be used to evaluate one protein pair. However, we will soon be developing a newer online version able to predict large numbers of PPIs.

## Figures and Tables

**Table 1.** Results of DFPCA and AAID with PRBF SVM in 10 CV test.

Feature Set	*S_n_* (%)	*PPV* (%)	*ACC* (%)	*MCC*
Hf	95.94 ± 1.92	91.98 ± 2.88	93.78 ± 1.44	0.8765
Vf	95.66 ± 2.75	92.52 ± 2.40	93.96 ± 1.86	0.8798
Pf	95.78 ± 2.23	92.07 ± 1.69	93.76 ± 1.93	0.8760
Zf	96.06 ± 1.24	91.71 ± 3.13	93.69 ± 1.86	0.8747
LEWP710101	95.86 ± 2.23	92.08 ± 4.32	93.80 ± 2.42	0.8768
QIAN880138	96.06 ± 2.83	92.27 ± 1.50	94.00 ± 1.22	0.8808
NADH010104	95.82 ± 2.98	92.04 ± 2.51	93.76 ± 1.66	0.8760
NAGK730103	96.06 ± 2.83	92.09 ± 4.02	93.90 ± 3.31	0.8789
AURR980116	95.94 ± 2.07	92.33 ± 1.42	93.98 ± 1.24	0.8804

**Table 2.** Results of RBF and PRBF with DFPCA in the 10 CV test.

Feature Set	Kernel Function	*S_n_* (%)	*PPV* (%)	*ACC* (%)
Hf	RBF	89.96 ± 0.52	89.65 ± 2.17	89.88 ± 1.05
	PRBF	95.94 ± 1.92	91.98 ± 2.88	93.78 ± 1.44
Vf	RBF	90.20 ± 1.31	89.33 ± 2.60	89.72 ± 1.72
	PRBF	95.66 ± 2.75	92.52 ± 2.40	93.96 ± 1.86
Pf	RBF	89.32 ± 0.86	89.26 ± 2.91	89.28 ± 1.44
	PRBF	95.78 ± 2.23	92.07 ± 1.69	93.76 ± 1.93
Zf	RBF	90.84 ± 1.85	88.79 ± 2.50	89.64 ± 1.18
	PRBF	96.06 ± 1.24	91.71 ± 3.13	93.69 ± 1.86

**Table 3.** Results of DF and DFPCA with PRBF SVM in the 10 CV test.

Feature Set	Feature Extraction Approach	*S_n_* (%)	*PPV* (%)	*ACC* (%)	*MCC*
Hf	DF	97.37 ± 2.55	66.67 ± 27.8	74.34 ± 24.3	0.5485
	DFPCA	95.94 ± 1.92	91.98 ± 2.88	93.78 ± 1.44	0.8765
Vf	DF	97.21 ± 2.39	71.40 ± 23.0	78.17 ± 27.1	0.6093
	DFPCA	95.66 ± 2.75	92.52 ± 2.40	93.96 ± 1.86	0.8798
Pf	DF	97.13 ± 4.70	69.48 ± 25.5	77.23 ± 27.2	0.5937
	DFPCA	95.78 ± 2.23	92.07 ± 1.69	93.76 ± 1.93	0.8760
Zf	DF	97.65 ± 4.82	62.29 ± 29.5	69.26 ± 23.6	0.4680
	DFPCA	96.06 ± 1.24	91.71 ± 3.13	93.69 ± 1.86	0.8747

**Table 4.** Effect of random sampling of the noninteracting protein subchain pairs on the performance of PPI-PKSVM with DFPCA and PRBF SVM in the 10CV test.

Sampling Time	*S_n_* (%)	*PPV* (%)	*AAC* (%)	*MCC*
1	95.38 ± 3.35	91.20 ± 3.37	93.09 ± 3.45	0.8627
2	95.42 ± 1.39	91.52 ± 3.24	93.29 ± 1.65	0.8665
3	95.46 ± 3.03	91.21 ± 1.63	93.13 ± 2.29	0.8635
4	95.46 ± 3.03	91.49 ± 1.70	93.29 ± 2.13	0.8666
5	95.94 ± 1.92	91.98 ± 2.88	93.78 ± 1.44	0.8765

**Table 5.** Performance comparison of different PPI methods using Shen’s dataset a in the 10 CV test.

Method	*S_n_* (%)	*S_p_* (%)	*ACC* (%)	*MCC*
LEWP710101	97.3 ± 0.04	99.2 ± 0.04	98.3 ± 0.00	0.966 ± 0.0006
QIAN880138	97.3 ± 0.10	99.1 ± 0.10	98.3 ± 0.10	0.966 ± 0.002
NADH010104	97.2 ± 0.07	99.2 ± 0.04	98.3 ± 0.05	0.965 ± 0.0007
NAGK730103	97.2 ± 0.06	99.2 ± 0.04	98.2 ± 0.06	0.965 ± 0.0004
AURR980116	97.3 ± 0.04	99.1 ± 0.06	98.2 ± 0.06	0.965 ± 0.0006
Hf-DFPCA	97.6 ± 0.20	99.1 ± 0.10	98.4 ± 0.10	0.967 ± 0.002
Vf-DFPCA	97.5 ± 0.10	98.9 ± 1.00	98.3 ± 0.80	0.965 ± 0.007
Pf-DFPCA	96.9 ± 0.10	99.5 ± 0.60	98.2 ± 0.60	0.964 ± 0.004
Zf-DFPCA	97.9 ± 0.90	96.0 ± 0.20	96.9 ± 1.10	0.939 ± 0.002
LDA-RF b	94.2 ± 0.40	98.0 ± 0.30	96.4 ± 0.30	0.928 ± 0.006
LDA-RoF b	93.7± 0.50	97.6 ± 0.60	95.7 ± 0.40	0.918 ± 0.007
LDA-SVM b	89.7 ± 1.30	91.5 ± 1.10	90.7 ± 0.90	0.813 ± 0.018
AC-RF b	94.0 ± 0.60	96.6 ± 0.40	95.5 ± 0.30	0.914 ± 0.007
AC-RoF b	93.3 ± 0.70	97.1 ± 0.70	95.1 ± 0.60	0.910 ± 0.009
AC-SVM b	94.0 ± 0.60	84.9 ± 1.70	89.3 ± 0.80	0.792 ± 0.014
PseAAC-RF b	94.1 ± 0.90	96.9 ± 0.30	95.6 ± 0.40	0.912 ± 0.007
PseAAC-RoF b	93.6 ± 0.90	96.7 ± 0.40	95.3 ± 0.50	0.907 ± 0.009
PseAAC-SVM b	89.9 ± 0.70	92.0 ± 0.40	91.2 ± 0.4	0.821 ± 0.006

a Shen’s dataset contains two subdatasets, C and D, which are available at http://www.csbio.sjtu.edu.cn/bioinf/LR_PPI/Data.htm;

b These results are taken from Table 4 of the literature [25].

**Table 6.** Amino acid groups classified according to their physicochemical value.

Physicochemical property	Group 1	Group 2	Group 3
Hydrophobicity	*H*_1_: R,K,E,D,Q,N	*H*_2_: G,A,S,T,P,H,Y	*H*_3_: C,V,L,I,M,F,W
van der Waals volume	*V*_1_: G,A,S,C,T,P,D	*V*_2_: N,V,E,Q,I,L	*V*_3_: M,H,K,F,R,Y,W
Polarity	*P*_1_: L,I,F,W,C,M,V,Y	*P*_2_: P,A,T,G,S	*P*_3_: H,Q,R,K,N,E,D
Polarizability	*Z*_1_: G,A,S,D,T	*Z*_2_: C,P,N,V,E,Q,I,L	*Z*_3_: K,M,H,F,R,Y,W
